# In vitro evaluation of a new balloon design to treat calcified stenosis

**DOI:** 10.1186/s42155-026-00662-2

**Published:** 2026-02-28

**Authors:** Oliver Andrew Binkert, Christoph Andreas Binkert, Thomas Frauenfelder

**Affiliations:** 1 Faculty of Medicine, University of Zurich, Forchstrasse 211, Zurich, ZH 8032 Switzerland; 2 Medizinisch Radiologisches Institut (MRI) Zurich, Zurich, ZH Switzerland; 3 Institute of Diagnostic Radiology, University Hospital Zurich, Zurich, ZH Switzerland

**Keywords:** Peripheral artery disease, Percutaneous transluminal angioplasty, New technology, In vitro, Below the knee (BTK)

## Abstract

**Background:**

Standard percutaneous transluminal angioplasty (PTA) occasionally falls short in sufficiently treating calcified lesions in below the knee arteries.

To address this, a new balloon design, called WAVE, was developed. This balloon is divided into multiple smaller segments, thereby creating a segment-waist shaped stress pattern. In combination with a procedural adjustment, involving three inflation cycles and a slight shift of the balloon between each inflation, the WAVE concept is proposed, in which enhanced fragmentation of calcified lesions is hypothesized.

**Purpose:**

A comparison between the standard of care PTA and the WAVE concept was made to evaluate the potential benefit of implementing the WAVE concept.

**Materials and methods:**

The experiment was evaluated using a calcified arterial in vitro model. The primary endpoint was to assess and compare both the total number of fragments and the number of fragments larger than 9 mm generated by the WAVE concept (including three inflations) versus the standard PTA (standard balloon with a single inflation).

**Results:**

The WAVE concept generated a significantly higher total number of fragments (112.6; 95% CI [103.3, 121.9]) compared to the standard balloon after a single inflation (48.6; 95% CI [42.5, 54.7]) (*p* < 0.001). The number of fragments exceeding 9 mm was significantly lower with the WAVE concept (7.6; 95% CI [6.4, 8.8]) compared to standard PTA (13.2; 95% CI [11.6, 14.8]) (*p* < 0.001).

**Conclusion:**

The WAVE concept creates more and smaller fragments in a below-the-knee calcified arterial in vitro model. These findings show promising potential to improve the outcomes of future angioplasty procedures.

## Background

Peripheral arterial disease (PAD) is an important health care issue. The following factors have been identified as contributors to the development of PAD: age, smoking, hypertension, renal disease, hyperlipidemia and diabetes [1]. As life expectancy has increased in the last years [2] and the prevalence of unhealthy lifestyle remains high [3], the population is more prone to PAD and its associated complications [4]. A pathological consequence of PAD is the calcification of arteries, with narrowing of the vascular lumen, significantly reducing blood flow and leading to ischemia in the affected area [5].

This study addresses the below the knee (BTK) arteriopathy which can lead to chronic limb-threatening ischemia (CLTI) [6]. BTK stenosis is especially common among diabetic patients. A lack of adequate blood circulation can result in the development of an ischemic foot, which must be treated to prevent the limb loss [7].

Arteries are composed of three layers: the tunica intima, the tunica media, and the tunica adventitia. The tunica intima is responsible for the formation of the superficial endothelial layer, which covers the subendothelial structures.

The tunica media is composed of numerous layers of vascular smooth muscle cells and forms a thick layer. These cells are responsible for regulating the diameter of the vessels by contracting or relaxing, thereby adjusting the blood flow.

The tunica adventitia, located externally, is composed of connective tissue, including collagen and elastic fibers, as well as an array of other structures, including vessels, nerves and lymphatic vessels [8].

It was found that, in contrast to iliac and femoral arteries where calcification typically occurs in the intimal layer, the calcifications in BTK arteries are more frequently observed in the tunica media, known as medial arterial calcification (MAC) [9]. With regard to the circumferential distribution, it was observed that calcification in the tunica media forms a more annular distribution, while calcification in the tunica intima accumulates in a non-circular, focal fashion [10].

In brief, the pattern of calcification in the BTK area is an annular calcification located in the tunica media. It was found that the annular composition of calcification results in more severe health issues compared to calcifications located in a focal manner [10].

Percutaneous transluminal angioplasty (PTA) is the most commonly used treatment for arterial stenoses. In this procedure, a balloon, which is placed to the stenotic lesion over a guidewire, is inflated to mechanically dilate the vessel by applying hydraulic pressure.

The most critical element of angioplasty is the successful establishment of adequate patency in the treated vessel. It is hypothesized that an increase in fragmentation of calcified lesions will result in improved outcomes.

Intravascular lithotripsy (IVL) addressed this aspect. This technique relies on different energy sources e.g. the use of ultrasound shockwaves to mechanically disrupt the calcified lesion wall and thereby generate a greater number of smaller, more granular fragments. IVL has shown promising results [11].

It is hypothesized that large fragments formed after angioplasty are more prone to recoil to their previous position. The reassembling of the smaller fragments appears to be less likely. Consequently, the amount of large fragments was specifically taken into consideration in this study.

It is crucial to note that the area undergoing PTA treatment may also contain healthy vessel areas that could potentially be damaged during the procedure, e.g. through dogboning or uncontrolled plaque dilatation, by applying excessive arterial stress [12]. This may contribute to possible iatrogenic dissections [12] or restenosis due to potential intimal hyperplasia and/or arterial wall remodeling, which are major causes of failure in the context of PTA [13]. The following products have been developed to address these issues:

The Chocolate balloon (Medtronic, MN – USA), a Scoring balloon, uses a nitinol cage to generate an alternating pillow-groove shaped stress pattern, thereby providing a predefined and therefore more controlled stress pattern to the affected vessel wall resulting in a more controlled fragmentation of the lesion. Additionally, reduced dogboning and a more controlled plaque dilatation are achieved resulting in less stress on surrounding healthy vessel segments.

The Angiosculpt (Philips, Amsterdam—Netherlands), also a scoring balloon, featuring a helical nitinol cage, provides a more controlled plaque dilatation and shows reduced dogboning. Consequently, less stress is applied to healthy vessel segments.

An additional benefit of controlled plaque dilatation is the ability to confidently perform 1:1 vessel-balloon-sizing, which has been shown to reduce the number of residual stenoses following procedures compared to cases using undersized balloons [14].

**Fig. 1:**
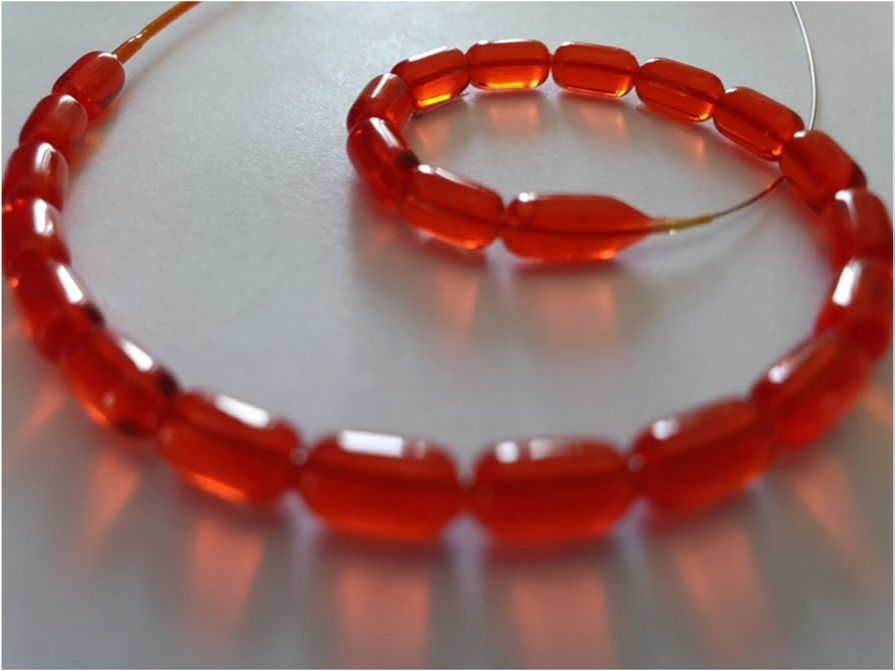
Image of the WAVE balloon

The new balloon design, called WAVE (Fig. 1), has been developed to improve the opening of these calcified vessels by increasing the fragmentation of the affected lesion due to its uniform design. The segment-waist design generates a predefined stress pattern, leading to a more controlled and enhanced fragmentation of the calcified lesion. It operates on a similar mechanism of action (MoA) to that of IVL, incorporating the concept of mechanically generating more and smaller fragments in a more controlled fashion.

### WAVE balloon

**Fig. 2:**
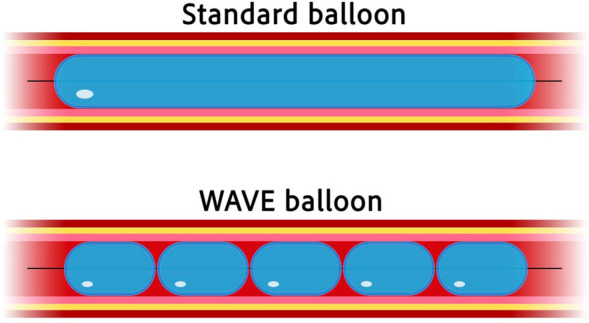
Scheme illustrating the structural differences between a standard balloon and the WAVE balloon

The main difference of the WAVE balloon compared to a standard angioplasty balloon is a segmentation into a series of short balloons (Fig. 2). The hypothesis is to fracture the calcified stenosis at multiple sites in a more controlled manner due to the predefined stress pattern, given by the balloons unique design, and thereby increasing fragmentation.

An additional hypothesis is that the reduced length of each segment will lead to decreased absolute longitudinal elongation during inflation. Given that longitudinal elongation during angioplasty can lead to vessel injury, the aim is to minimize this occurrence [15].

**Fig. 3:**
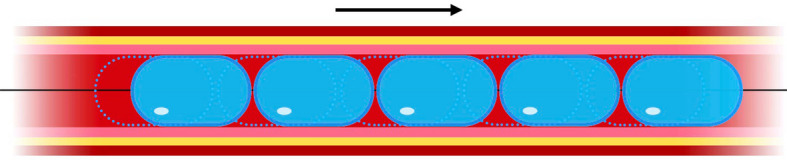
Scheme illustrating the effect of slightly shifting the balloon between the inflations. With this change in procedure, the stress pattern acting on the vessel wall can be repositioned after every inflation cycle

To further optimize the result a procedural adjustment is suggested by adding a 2nd and 3rd inflation cycle. After the first inflation cycle, the WAVE balloon is advanced by 3 mm and after the second inflation, it is retracted by 1.5 mm in order to shift the stress pattern acting on the vessel wall (Fig. 3).

With this change in design and procedure we suggest a new WAVE concept. The goal of this innovation is to enhance the fragmentation of calcified vessels, resulting in a greater number of fragments and a reduced number of large fragments. This should result in improved outcomes concerning long-term vessel patency.

### Endpoints overview

The *primary endpoint* of this in vitro experiment is to analyze the total number of fragments and the number of large fragments, defined as fragments exceeding 9 mm in length, after angioplasty. The experiment compares two procedures: the WAVE concept, which includes the WAVE balloon with three inflation cycles and a slight shift of the balloon after each inflation, and the standard PTA procedure, which includes a standard balloon with a single inflation, reflecting standard of care.

In order to differentiate the impact of the balloon design and the modified inflation procedure, the following experiments were additionally performed: the application of the WAVE balloon with a single inflation cycle only, and the application of a standard balloon with three inflation cycles including the shift after each inflation.

## Material and methods

To ensure the most accurate representation, the in vitro model must have comparable characteristics to the stenotic vessels observed in the BTK area in vivo.

### Diameter—in vitro model

The diameters of the three crural arteries (anterior tibial artery, posterior tibial artery, and fibular artery) range from 2.48 to 3.52 mm [16], which is the reason why a diameter of 3 mm was selected for the model in use for this study.

### Degree of stenosis—in vitro model

The specific degree of stenosis which has a significant hemodynamic impact remains somewhat unclear. According to the criteria established in other imaging studies, a stenosis of at least 50% is classified as significant and is therefore employed in our model [17]. The internal diameter of the in vitro stenosis was set to 50% of the vessel lumen, resulting in a diameter of 1.5 mm.

### Length – in vitro model

**Fig. 4:**
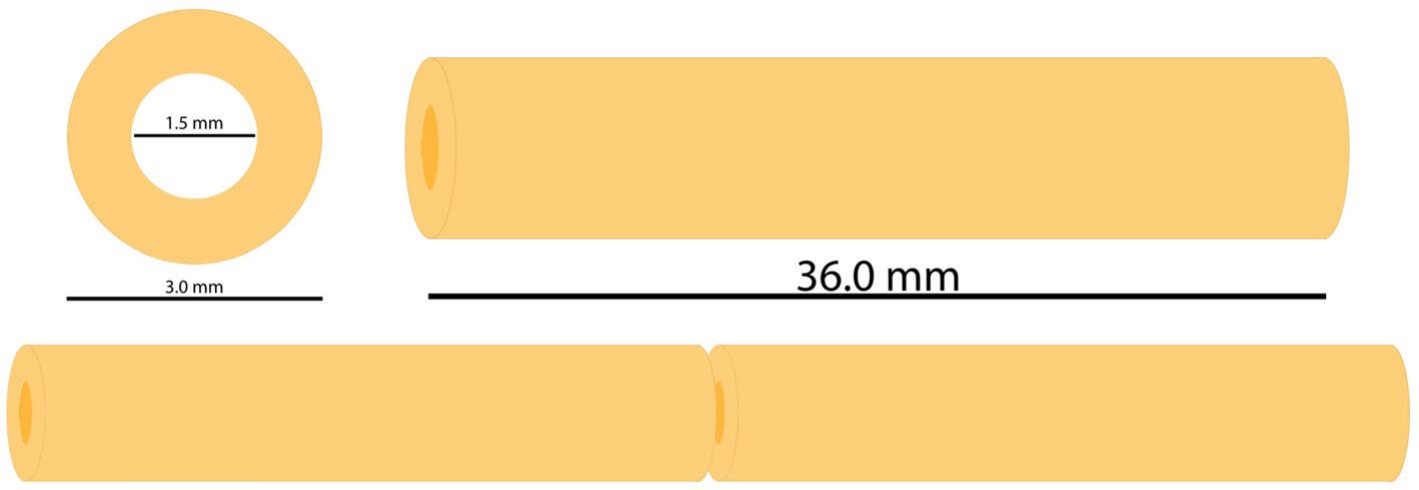
Scheme and dimensions of the in vitro model

The lengths of stenoses and occlusions in the BTK region vary considerably but are generally longer than those found in the femoropopliteal arteries. A study focusing on diabetic patients reported that more than 50% of stenoses in the anterior and posterior tibial arteries exceeded 10 cm in length [7]. Lesions in the fibular artery were most commonly between 5 and 10 cm [7]. Another study found that the average lesion length in infrapopliteal vessels was 7.3 ± 6.3 cm [15]. In our in vitro model, the total vessel length was set to 7.2 cm. Since such lesions typically do not extend continuously over the entire vessel segment, the model consisted of two separate 3.6 cm sections (Fig. 4).

### Material selection – in vitro model

Arterial lesions contain variable amounts of relatively soft and fatty lipids, fibrous tissue, and hard, calcified material. The degree of calcification correlates with the presence of such calcium deposits within the arterial wall. For an accurate modeling of the in vitro model, when considering the fracture behavior, it is necessary to know the mechano-elastic properties of each layer in the diseased tissue, as well as their distribution within the lesion wall.

The first step was to determine the elastic modulus (E-modulus) of each layer in MAC lesions. The E-modulus is a measure of a material's stiffness or resistance to elastic deformation under stress (Elastic modulus; in [MPa]) [18]. Based on existing publications that provided data on the E-moduli of these layers in vivo (e.g., fibrous and calcified tissue) [19, 20], appropriate materials were selected that, in combination, reflect the range of elastic moduli observed in in vivo lesions.

The materials chosen for testing are listed in Table 1. To validate the suitability of this material mixture, the pressure required to fracture the in vitro model using a standard PTA balloon was measured and compared to the pressures typically needed to dilate stenotic vessels in vivo during PTA procedures. Two referenced studies report pressure ranges of 4–12 atm [21] and 4–8 atm [14], respectively. As the in vitro model required fracture pressures between 5 and 8 atm—within the clinical range—the material mixture was deemed suitable for testing.

**Table 1:** Materials and their composition selected for the in vitro model. The E-modulus describes a material's stiffness or its resistance to elastic deformation under stress

Material	E-modulus [MPA]	Composition %(w/w)
Paraffin wax^a^	38–313 [22]	66.6
Carnauba wax^b^	1806 [22]	16.6
Calcium Carbonate^c^	68.000–88.000 [23]	16.6

^a^granulated (CAS-No. 8002–74-2), melting point 55 °C

^b^type T1, flakes (CAS-No. 8015–86-9), melting point range 81–86 °C

^c^powdered (CAS-No. 471–34-1)

### Material preparation—in vitro model

Prior to preparation and testing, all materials were stored in a controlled environment at 22 ± 3 °C and 45 ± 10% humidity. The material mixture for artificial lesion formation was prepared by adding 40 ± 1 g paraffin wax, 10 ± 1 g carnauba wax, and 10 ± 1 g calcium carbonate to a 150 ml wide-necked glass bottle. After adding a magnetic stirrer and closing the bottle, the components were heated in a water bath with continuous stirring at over 85 °C until all components were homogeneously dispersed. The mass percent composition was adjusted to, as mentioned in the table above, 66.6 (w/w) paraffin wax, 16.6 (w/w) carnauba wax, and 16.6 (w/w) calcium carbonate (CaCO3).

### Molding – in vitro model

**Fig. 5:**
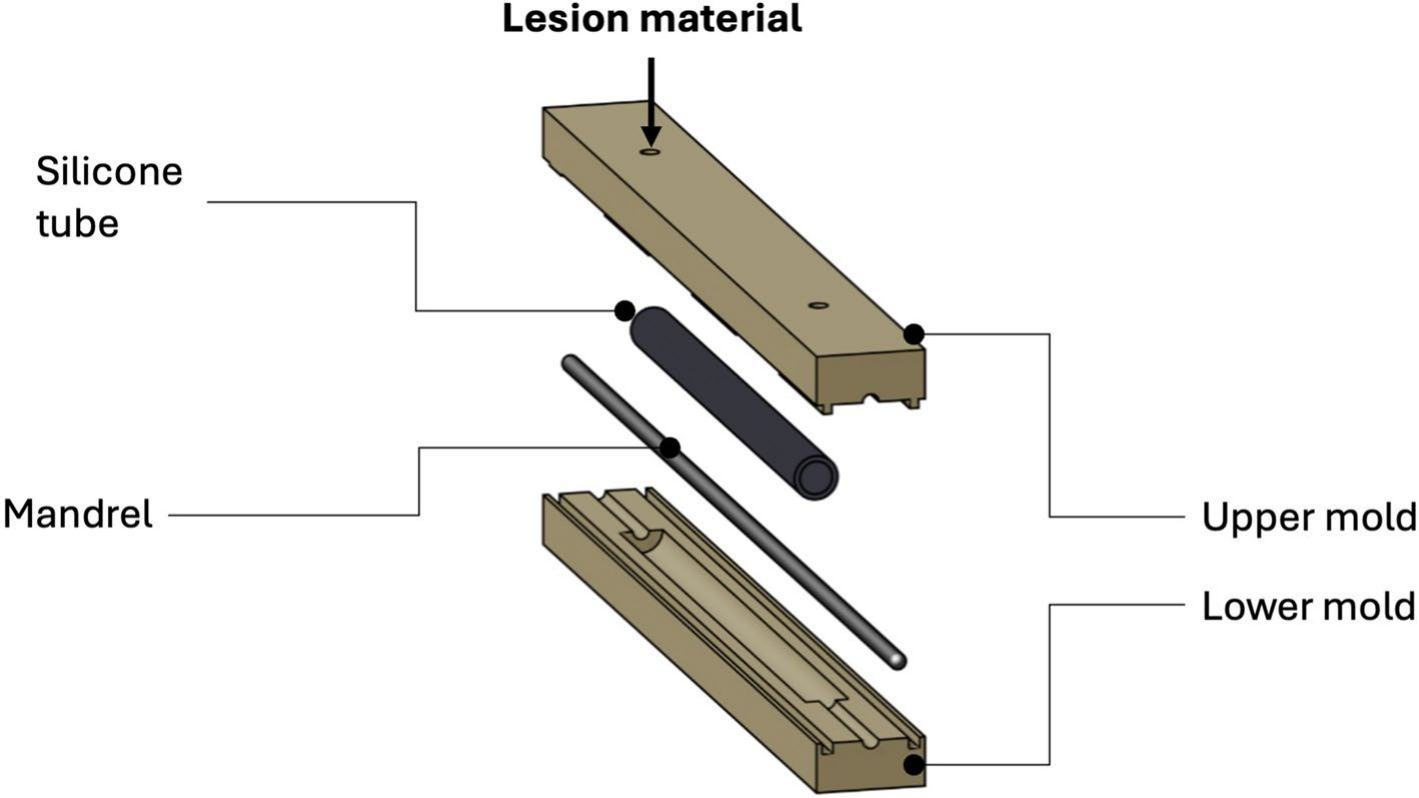
Scheme of the custom two-part mold used to cast the in vitro models

For lesion molding, a custom two-part mold was 3D-printed, as illustrated in Fig. 5. The mold featured a cylindrical injection cavity (outer diameter: 4.0 mm; length: 54 mm), into which a silicone tube (outer diameter: 4.0 mm; wall thickness: 0.5 mm; length: 52 mm) was placed. A mandrel (outer diameter: 1.5 mm) was inserted through the silicone tube, and the mold was subsequently closed. The liquefied and homogeneously mixed lesion material was drawn into a 10 ml syringe and immediately injected into the cavity. After injection, the mold was cooled in a laboratory refrigerator for 5 min. The artificial lesion was then carefully removed from the mold along with the mandrel and silicone tube. The silicone tube was longitudinally cut open with a razor blade and removed. Finally, the mandrel within the model was carefully removed.

The in vitro models were stored in a controlled environment for 7 to 9 days prior to testing.

For testing, the final model was assembled by placing two preformed lesion segments (each 3.6 cm in length) in series within a single silicone tube of 7.4 cm total length to prevent protrusion. This silicone tube had an internal diameter of 3 ± 0.1 mm (corresponding to the outer diameter of the stenosis) and an outer diameter of 4 mm. It featured a Shore A hardness of 60 ± 5 (2.2 MPa), simulating the mechanical resistance of a diseased arterial wall, typically represented by fibrous tissue with an approximate E-modulus of 1.3 MPa.

### Experiment

The experiment was conducted in a standardized manner using the WAVE balloon (3 mm × 80 mm) and a standard balloon (Bard Ultraverse 0.014: 3 mm × 80 mm). The balloon was centrally positioned within the in vitro model and gradually inflated to an initial pressure of 4 atm. Subsequently, the pressure was increased in 1 atm increments, with each pressure level maintained for 30 s. This stepwise inflation was based on the assumption that prolonged pressure application enhances the effect on the stenotic wall, potentially resulting in rupture at lower pressure levels. Lower pressure levels minimize excessive stress on surrounding healthy vessel segments.

If a pressure drop occurred due to a partial opening of the stenosis, the pressure was immediately readjusted to the target level. This process continued until the lesion was fully fractured. The balloon was then deflated and advanced by 3 mm to shift the stress pattern. The inflation procedure was repeated, this time inflating the balloon to its rated burst pressure (RBP) — 14 atm for the WAVE and 15 atm for the Bard Ultraverse. After deflation, the balloon was retracted by 1.5 mm, again to shift the stress pattern, and the procedure was repeated a third time.

Following the third inflation cycle, the balloon and guidewire were removed. The silicone tube was carefully opened using a razor blade, and the resulting fragments were collected and analyzed.

To evaluate the impact of the balloon design, the three-cycle procedure was performed ten times for each balloon type (*n* = 10). Additionally, to assess the effect of inflation frequency, a single-inflation experiment was conducted for both balloon types (*n* = 5).

### Contrast agent solution

A solution consisting of 50% 0.9%-sodium chloride (NaCl) and 50% contrast agent (Visipaque, 320 mg iodine/ml) was used. To visualize the mixture during the test, three drops of red colorant were added.

### Fragment analysis

The total number of fragments and the number of fragments exceeding 9 mm in length were counted. The fragments were positioned beneath a digital microscope (Keyence VHX 6000, Keyence Corporation, OSA—Japan) equipped with a camera, enabling precise counting and measurement.

Statistical analysis was performed using independent parametric two-sample t-tests. Since the study hypotheses were directional—specifically, an increase in the total number of fragments and a decrease in the number of fragments exceeding 9 mm in length when applying the WAVE concept—one-tailed t-tests were used. Corresponding to a 95% confidence level, *p*-values below 0.05 were considered statistically significant.

## Results

**Fig. 6:**
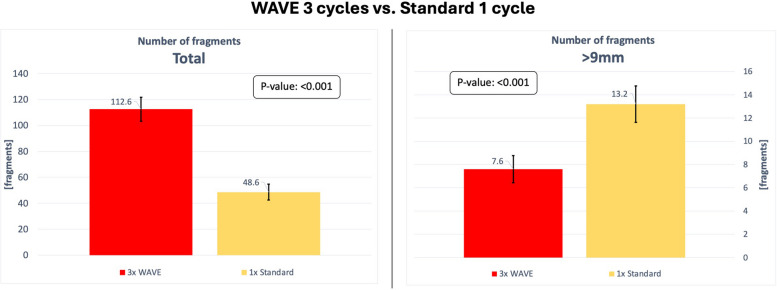
Chart comparing the results of the experiments WAVE 3× and Standard 1×. The diagram on the left compares the average total number of fragments. The diagram on the right compares the average number of fragments exceeding 9 mm in length. The 95% confidence interval is indicated for each value

The total number of fragments, as well as the number of fragments exceeding 9 mm, are listed in Table 2. The primary endpoint of the study, demonstrated in Fig. 6, was to compare the standard of care PTA procedure, using a standard balloon with one inflation cycle, to the WAVE concept, which contains the WAVE balloon and three inflation cycles and a shift of the balloon after each inflation:

**Table 2:** The results of all experiments. It counts the total number of fragments, the number of fragments exceeding 9 mm, and provides the averages

WAVE 3×	n	1	2	3	4	5	6	7	8	9	10	⌀
Total number of fragments		141	111	120	86	100	110	108	109	128	113	112.6
Number of fragments > 9 mm		6	7	5	10	9	6	7	11	7	8	7.6
Standard 3×	n	1	2	3	4	5	6	7	8	9	10	⌀
Total number of fragments		91	78	87	80	77	82	94	92	122	84	88.7
Number of fragments > 9 mm		13	14	11	14	12	14	17	10	10	11	12.6
WAVE 1×	n	1	2	3	4	5	⌀					
Total number of fragments		47	43	22	50	30	38.4					
Number of fragments > 9 mm		6	11	11	14	14	11.2					
Standard 1×	n	1	2	3	4	5	⌀					
Total number of fragments		57	47	51	38	50	48.6					
Number of fragments > 9 mm		13	16	13	11	13	13.2					

WAVE 3×: WAVE balloon was used with three inflation cycles, including a slight shift between each inflation. (*n* = 10)

Standard 3×: Standard balloon was used with three inflation cycles, including a slight shift between each inflation. (*n* = 10)

WAVE 1×: WAVE balloon was used with one inflation cycle. (*n* = 5)

Standard 1×: Standard balloon was used with one inflation cycle. (*n* = 5)

The average total number of fragments when the WAVE balloon was used for three cycles was 112.6 (95% CI [103.3, 121.9]). The average total number of fragments when the standard balloon was used for a single cycle was 48.6 (95% CI [42.5, 54.7]). These findings show a highly significant increase in the number of fragments when the WAVE concept was performed (*p* < 0.001).

The average number of fragments exceeding 9 mm when the WAVE balloon was used for three cycles was 7.6 (95% CI [6.4, 8.8]). The average number of fragments exceeding 9 mm when the standard balloon was used for a single inflation cycle was 13.2 (95% CI [11.6, 14.8]). These findings show a significant reduction in the number of fragments exceeding 9 mm when the WAVE concept was performed (*p* < 0.001).

**Fig. 7:**
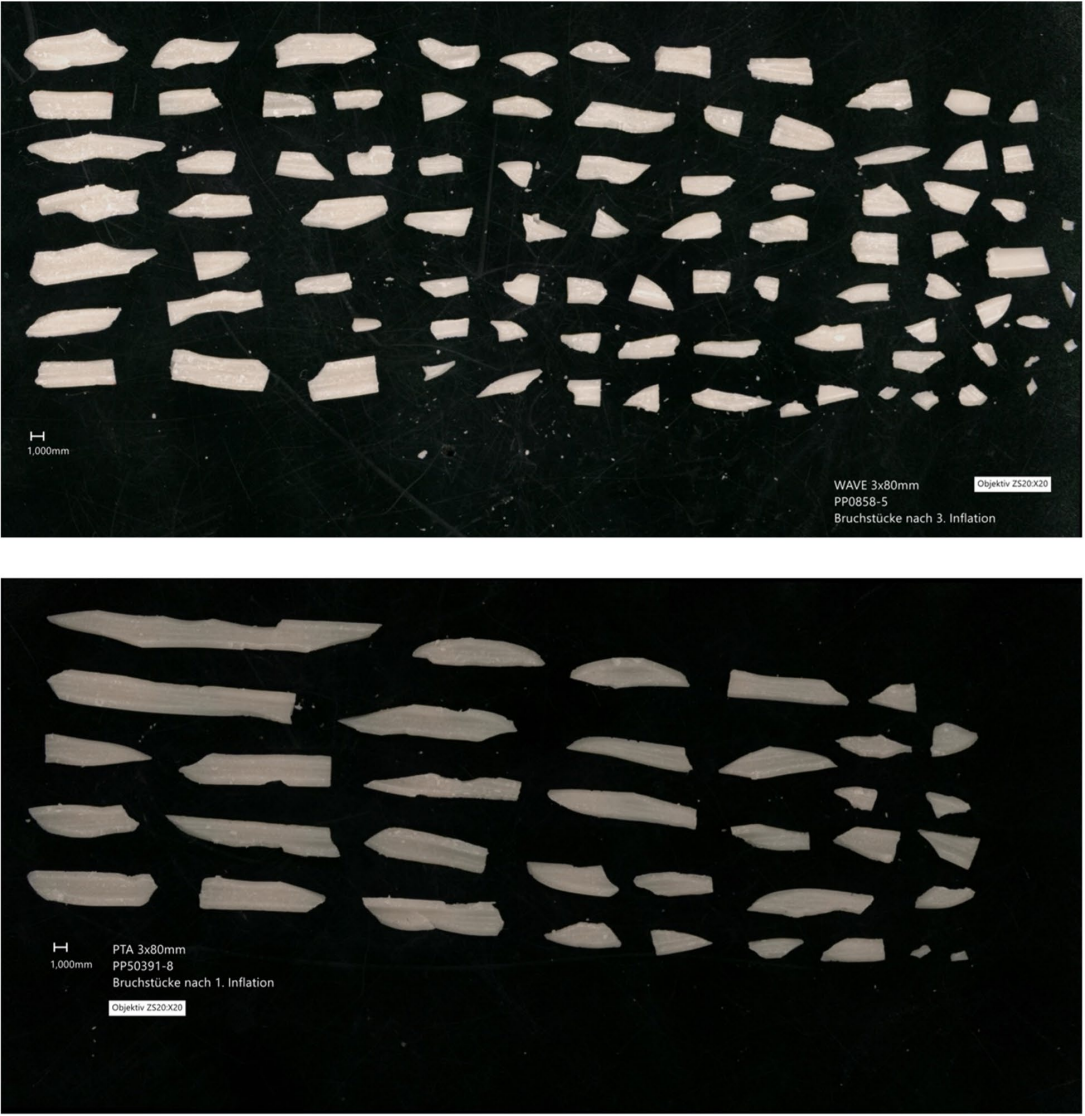
An illustrative comparison of the fragments generated after three cycles with the WAVE balloon (top image) and one cycle with the standard balloon (bottom image). The images include a 1 mm scale

Figure 7 shows an illustrative comparison of the fragments after three cycles with the WAVE balloon (top image) and one cycle with the standard balloon (bottom image). The difference in number and size is apparent.

**Fig. 8:**
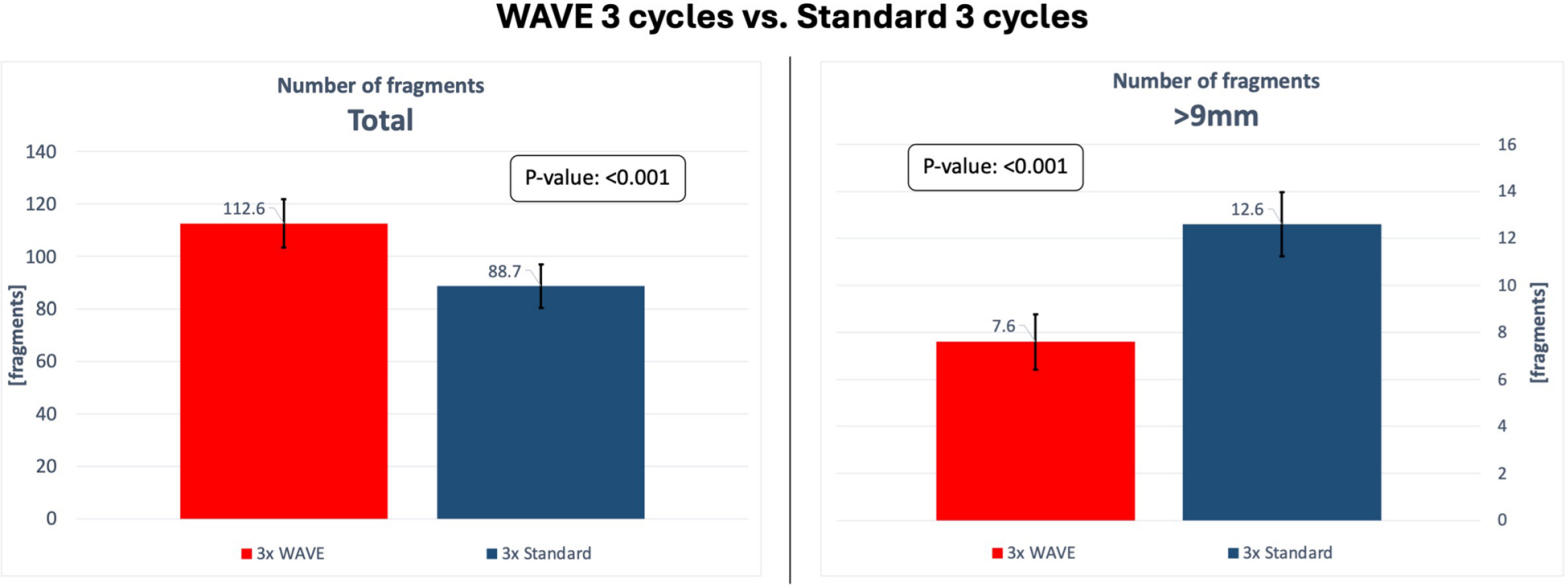
Chart comparing the results of the experiments WAVE 3× and Standard 3×. The diagram on the left compares the average total number of fragments. The diagram on the right compares the average number of fragments exceeding 9 mm in length. The 95% confidence interval is indicated for each value

In order to show the impact of the balloon design only, the outcomes of the WAVE balloon and standard balloon following three inflation cycles including the shift after each cycle were evaluated and demonstrated in Fig. 8. The WAVE balloon showed 112.6 fragments (95% CI [103.3, 121.9]), the standard balloon 88.7 fragments (95% CI [80.6, 96.8]) on average. This difference is statistically significant (*p* < 0.001).

With the WAVE balloon, 7.6 fragments (95% CI [6.4, 8.8]) exceeding 9 mm in length were observed, while with the standard balloon, 12.6 fragments (95% CI [11.2, 14.0]) exceeding 9 mm in length were counted on average. This result shows a significant decrease in the number of large fragments (> 9 mm) when using the WAVE balloon (*p* < 0.001).

**Fig. 9:**
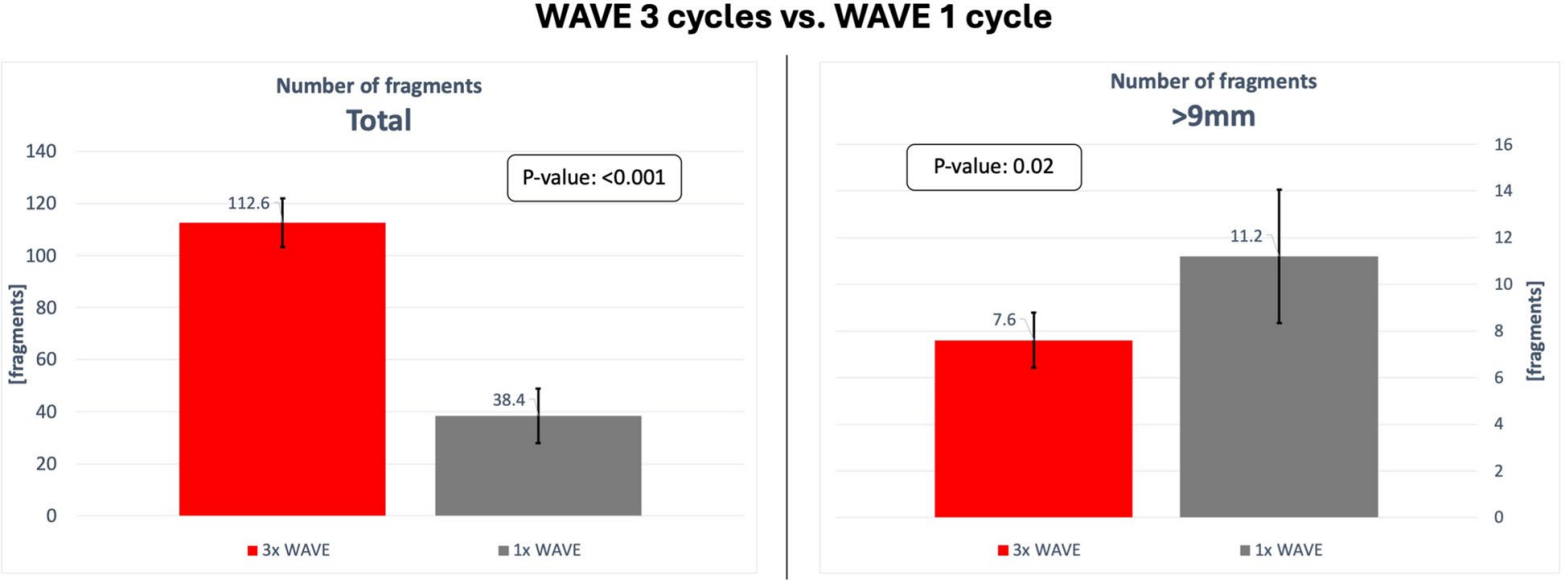
Chart comparing the results of the experiments WAVE 3× and WAVE 1×. The diagram on the left compares the average total number of fragments. The diagram on the right compares the average number of fragments exceeding 9 mm in length. The 95% confidence interval is indicated for each value

In order to evaluate the impact of the modified procedure, a comparative analysis was performed on both the WAVE and the standard balloon:

Figure 9 illustrates the results comparing the number of inflations using the WAVE balloon. The average total number of fragments, when the WAVE balloon was used for a single inflation, was only 38.4 (95% CI [27.9, 48.9]) indicating a statistically significant increase in the number of fragments when the procedure was continued with three inflation cycles in total (*p* < 0.001).

The average number of fragments exceeding 9 mm after a single cycle using the WAVE balloon was 11.2 (95% CI [8.3, 14.1]). The average number of large fragments was reduced by approximately 38% when three inflations were performed, indicating a statistically significant difference (*p* = 0.02).

**Fig. 10:**
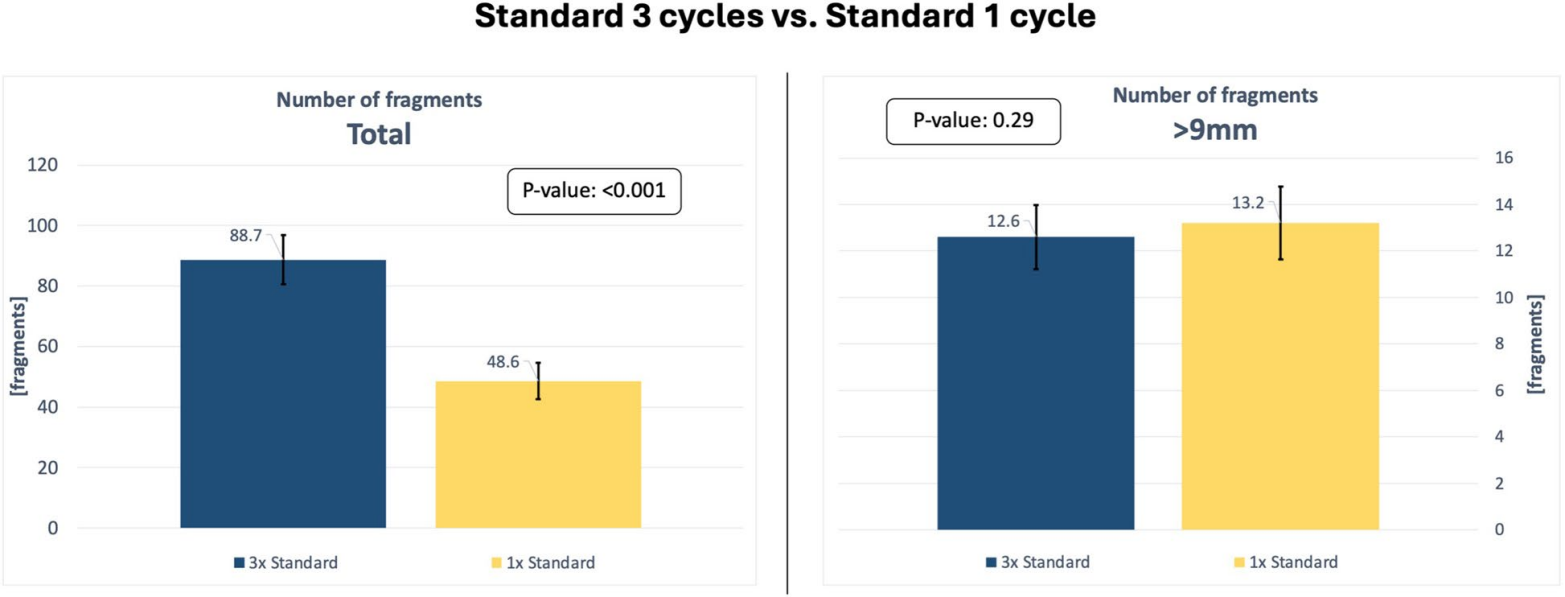
Chart comparing the results of the experiments Standard 3× and Standard 1×. The diagram on the left compares the average total number of fragments. The diagram on the right compares the average number of fragments exceeding 9 mm in length. The 95% confidence interval is indicated for each value

Figure 10 illustrates the results comparing the number of inflations using the standard balloon. When the standard balloon was used for a single cycle, the average total number of fragments was 48.6 (95% CI [42.5, 54.7]), while after three cycles with the standard balloon it was 88.7 (95% CI [80.6, 96.8]). This finding indicates a statistically significant increase in the number of fragments when the procedure was continued with three cycles in total (*p* < 0.001).

However, the average number of fragments exceeding 9 mm following a single cycle with the standard balloon was 13.2 (95% CI [11.6, 14.8]), and after three cycles, it was 12.6 (95% CI [11.2, 14.0]). These results show no statistically significant difference between the number of large fragments (> 9 mm) observed following one and three inflation cycles (*p* = 0.29).

## Discussion

CTLI, ischemia of the lower limbs due to severely calcified stenoses in the BTK area, is a serious healthcare issue. The calcifications in the BTK area predominantly manifest in a MAC configuration, an annular calcification of the tunica media. Standard angioplasty with a standard balloon occasionally fails to treat these calcified stenoses successfully. The excessive arterial stress applied to the vessel through dogboning and uncontrolled plaque dilatation, including the healthy vessel areas surrounding the lesion, may lead to dissections or restenosis of the vessel, resulting in unfavorable outcomes.

A new balloon design, called WAVE, has been proposed. The WAVE balloon is divided into a series of short balloon segments and waists. The MoA of segmental inflation leads to an application of a predefined stress pattern to the lesion wall, thereby inducing enhanced controlled mechanical fragmentation into smaller and more granular fragments. It has been demonstrated that the fragmentation process can be significantly amplified by shifting the balloon by a few millimeters following each inflation cycle.

With regard to the primary endpoint, it was found that the proposed WAVE concept produced a higher total number of fragments and fewer fragments > 9 mm compared to the standard of care PTA procedure, including one inflation.

In addition to the new WAVE design, the modified procedure with repeated inflation and positional shift of the balloon led to a significant increase in the total number of fragments and the number of fragments > 9 mm when the WAVE balloon was used. On the other hand, three inflations with the standard balloon only increased the total number of fragments, but did not reduce the number of fragments > 9 mm.

When comparing the results using the WAVE balloon with only a single inflation cycle and three inflation cycles, a highly significant benefit for three inflation cycles was observed. Therefore, it is crucial to note that the strength of the WAVE balloon design is maximized when performing multiple inflation cycles.

The concept of mechanically creating more and smaller fragments in calcified lesions has been applied by IVL with promising results [11]. It is expected that creating smaller, more granular fragments will reduce the number of recoil events, where the generated fragments return to their previous position, compared to larger fragments. This would result in better vessel patency. While the WAVE balloon appears to demonstrate a comparable effect in vitro, further research is necessary to substantiate these findings in vivo.

In conclusion, the WAVE concept, consisting of a new, multi-segmented balloon design and a procedural adjustment of three inflation cycles, including a slight shift of the balloon, creates more and smaller fragments in a BTK calcified arterial in vitro model compared to the standard of care PTA procedure. These promising results have the potential to improve future revascularization of BTK calcified lesions.

## Limitations

The experiment and its corresponding results were conducted in an in vitro model. The in vitro model demonstrates an ideal cylindrical calcification of the tunica media. However, in vivo lesions in the BTK area present with variable circumferential distributions and demonstrate calcification predominantly in the tunica media, but not exclusively. Furthermore, the forces associated with blood flow have not been incorporated into the model.

The endpoints of this study focus on the fragmentation of the calcified lesion. The in vitro model used did not allow assessment of the luminal gain nor vessel compliance.

Reduced balloon elongation with the WAVE design and, consequently, reduced occurrence of vessel injury is only hypothesized and was not demonstrated in this study.

For those reasons, these results must be confirmed in future in vivo models.

## Data Availability

All data generated or analyzed during this study are included in this published article.

## Abbreviations

PTA: Percutaneous transluminal angioplasty
PAD: Peripheral arterial disease
BTK: Below the knee
CLTI: Chronic limb-threatening ischemia
MAC: Medial arterial calcification
IVL: Intravascular lithotripsy
MoA: Mechanism of action
RBP: Rated burst pressure
